# Imaging-Based Biomarkers: Characterization of Post-Kawasaki Vasculitis in Infants and Hypertension Phenotype in Rat Model

**DOI:** 10.1155/2012/364145

**Published:** 2012-06-14

**Authors:** Roch Listz Maurice, Nagib Dahdah, Johanne Tremblay

**Affiliations:** ^1^Centre de Recherche, Centre Hospitalier de l'Université de Montréal (CRCHUM), Université de Montréal, Montréal, QC, Canada; ^2^Division de la Cardiologie Pédiatrique, Centre Hospitalier Universitaire Ste Justine (CHUSJ), Université de Montréal, Montréal, QC, Canada; ^3^Centre de Recherche, Centre Hospitalier Universitaire Ste Justine (CRCHUSJ), Université de Montréal, Montréal, QC, Canada; ^4^Laboratoire de Biologie Cellulaire de l'Hypertension, CRCHUM, Université de Montréal, Montréal, QC, Canada; ^5^Faculté de Médecine, Université de Montréal, Montréal, QC, Canada

## Abstract

*Background*. Investigating the mechanical properties of the arteries is essential in cardiovascular diseases. Recent imaging modalities allow mapping mechanical properties within the arterial wall. *Aims*. We report the potential of *imaging-based biomarker* (ImBioMark) to investigate the effect of aging on the rat. We also present preliminary data with ImBioMark characterizing vascular sequelae of Kawasaki disease (KD) in young humans. *Methods*. We investigated *in vivo* the effect of aging on male Brown Norway (BN) rats' (*n* = 5) carotid stiffness. In a second experiment, the impact of KD on the ascending aorta (AA) was examined in KD children (*n* = 2) aged 13 ± 1.41 years old compared to KD-free children (*n* = 5) aged 13.13 ± 0.18 years old. *Results*. The stiffness of BN's carotid artery was three times stiffer in the old rats, with a turning point at 40 weeks old (*P* = 0.001). KD had a very significant impact on the AA stiffness with strain estimates of 2.39 ± 0.51% versus 4.24 ± 0.65% in controls (*P* < 0.001). *Conclusion*. ImBioMark phenotypes hypertension in rat models noninvasively *in vivo* without resorting to euthanasia. Quantifying aortic wall remodeling is also feasible in humans. Future investigations target human cardiovascular disease.

## 1. Introduction

Age-related stiffening of large arteries reduces their function of transforming a pulsatile to a more even blood flow; this may be the most serious change affecting the cardiovascular system [1, 2]. Accordingly, arterial stiffness has been reported to be an independent predictor of cardiovascular mortality [3]. Provided there is progressive fibrosis of large arteries that reduce their elasticity, aging is known to be the dominant process altering arterial stiffness. This stiffness alteration with age is accelerated in hypertension and amplified by the association with other cardiovascular diseases.

On the other hand, Kawasaki disease (KD) is an immune-mediated pathology that mostly affects children under 5 years old, and in which multiorgan vasculitis affects large and mid size arteries. KD was described in 1967 by Tomisaku Kawasaki as a self-resolving disease causing acute vasculitis [4, 5]. The prognosis of the disease largely depends on the extent of coronary artery (CA) complications that may lead to myocardial infarction and possibly to death [6, 7]. Nevertheless, the reported dilatation of the ascending aorta (AA) along with aneurismal formation in major arteries, such as the axillary, carotid, renal, celiac, and femoral arteries to name a few [8], compelled us to address potential remodelling of the AA. Our initial experience involved 64 KD patients, among whom 15 had CA aneurysms, and 154 healthy children aged 9.7 ± 6.1 and 7.9 ± 5.1 years, respectively (*P* = 0.39) [9]. The methodology that was used in that work utilized equations conceived for invasive measurements and adapted for noninvasive ultrasound derived data [10]. Accordingly, significant alterations were observed in the elastic pressure strain modulus, the *β* resistance index, as well as the distensibility. On this basis, we are planning to perform direct measurements from the AA wall motion and characteristics. The methodology we expose in the current paper represents the working template of our group.

In addition, mechanical properties of biological soft tissues can be used as a diagnostic tool and potentially as an element for prognostic estimates as well. With this regards, Non-Invasive Vascular Elastography (NIVE) was proposed to characterize superficial arteries [11]. We recently addressed the feasibility and reproducibility of NIVE for the purpose of *in vivo* applications in human carotid arteries [12]. In that clinical trial, we derived an elastic modulus calculation that, on average, exhibited less than 100 kPa for the population free of vascular disease. Data were reported for the left and right common and internal carotid arteries, respectively.

NIVE has been extended to investigate small vessel walls in the order 150 *μ*m in rodent models of hypertension, which was labelled as MicroNIVE [13]. In a very recent study, we observed that female recombinant inbred hypertensive rat model (RI-17) carotid stiffness increased with age by a factor of about 2 between the age of 30 and 80 weeks old [14]. In that same paper, we also reported preliminary data on the impact of various salt diets on the carotid stiffness in different hypertensive rat models. Accordingly, a high salt diet induces an increase in BP, which could subsequently produce the carotid artery wall remodeling only in a mid- or long-term perspective. MicroNIVE can, therefore, provide reliable mechanical markers for vascular tissue investigations. We herein refer to the technique as an Imaging-based BioMarker (ImBioMark).

This paper reports additional data of ImBioMark that confirm the effect of aging on the rat carotid artery stiffness. The common carotid artery of male Brown Norway (BN) rats (*n* = 5) was investigated *in vivo* and throughout the animals' lifetime, that is, from 15 up until 105 weeks old. We also report preliminary feasibility data supporting the potential of ImBioMark to investigate human vascular sequalae as we study the impact of KD on the AA.

## 2. Methodology

### 2.1. Hypertension Phenotype in Rat Models

#### 2.1.1. Animal Models

The present investigation conforms to Canadian Council on Animal Care (CCAC) guidelines as well as the Guide for the Care and Use of Laboratory Animals published by the US National Institutes of Health (NIH Publication no. 85-23, revised 1996). All procedures were approved by the institutional Animal Care Committee of the *Centre hospitalier de l'Université de Montréal* (CHUM).

In this experiment, the left and right common carotid arteries of 5 male BN rats were studied *in vivo* and throughout animals' lifetime, that is, from 15 up until 105 weeks old. They were anaesthetized by inhalation of 1.5% isofluorane during ultrasound scanning. Body temperature was maintained at 37 ± 1°C by a heated surface and monitored with a rectal probe (Thermalert TH-5, Physitemp Instruments, Clifton, NJ, USA). Systolic blood pressure (SBP) was measured with a tail-cuff monitoring system (Model XBP-1000, Kent Scientific, Torrington, CT, USA); such pressure is indicative of mean systemic pressure of the animals [15]. Hair over the neck was shaved and further removed with depilatory cream (Nair, Church & Dwight Co., USA) to facilitate ultrasound recordings with a coupling gel.

#### 2.1.2. Experimental Setup

Time sequences, that is, dynamic radio-frequency (RF) images, were recorded, for typically 7 cardiac cycles (CC), over longitudinal segments of the arteries with the *Vevo 660* ultrasonic biomicroscope (Visualsonics, Toronto, ON, Canada). The *Vevo* was equipped with an encapsulated oscillating single element transducer. A 40-MHz central frequency probe (*f* number = 2, diameter = 3 mm, focal length = 6 mm, and fractional bandwidth at −6 dB = 110%) was used. The frame rate was 60 images/s. All data were digitized with an acquisition board (Model 8500 CS, Gagescope, Montreal, QC, Canada) installed in a personal computer. Sampling frequency was 500 MHz in an 8-bit format.

#### 2.1.3. Motion Estimation

The motion estimation method that is used to compute mechanical parameters from BN rats' carotid RF data is described in detail elsewhere [13]. In summary, it required partitioning the RF data into small measurement-windows (MW) in which the tissue motion is assumed to be affine between two consecutive images. More explicitly, let us consider an MW at time “*t*” (*I*(*x*(*t*), *y*(*t*))) and the same MW at time “*t* + *δt*” (*I*(*x*(*t* + *δt*), *y*(*t* + *δt*))). The method consists of computing the affine transformation that allows the best match between MW at “*t*” and “*t* + *δt*”, that is

(1) MINΔ⁡||I(x(t),y(t))−I(x(t+δt) −[T1],y(t+δt)−[T2])||2.

As a first step of the algorithm, lateral (*T*_1_) and axial (*T*_2_) translations are computed using cross-correlation technique. Following that, the 2D-deformation matrix (Δ) is then assessed through solving a non linear minimization problem. Δ can be expressed as

(2) Δ(t)=[Δxx(t)Δxy(t)Δyx(t)Δyy(t)],

where Δ_*xx*_ is the lateral strain, Δ_*yy*_ is the axial strain, and Δ_*xy*_ and Δ_*yx*_ are lateral and axial shear parameters, respectively. Provided the axial strain is a relative measure of stiffness, data are reported for only Δ_*yy*_ in the context of this paper.

Δ_*yy*_ was computed for each pair of consecutive RF images using MW of 108 × 312 *μ*m^2^ with 90% axial and lateral overlaps. A wall mean strain value (${\overset{-}{\Delta }}_{yy}$) was calculated from each Δ_*yy*_. Because of RF echo attenuation with depth that reduces the signal-to-noise ratio (SNR), strain measurements were performed only at the near wall.

### 2.2. Characterization of Post-KD Vasculitis

#### 2.2.1. The Pediatric Population

Data from a small sample (*n* = 2) of children diagnosed with KD during the preschool age were compared to samples obtained from healthy children (*n* = 5) of similar age at the time of this investigation (13 ± 1.41 years old versus 13.13 ± 0.18 years old, resp.). B-mode echocardiography video sequences were acquired in the left parasternal acoustic window at the second or third intercostal space using either a iE33 Philips (Philips, Eindhoven, the Netherlands) or a GE Vivid 8 (GE Healthcare, Louisville, Kentucky) echocardiograph. Images were focused on the ascending aorta at the point of intersection with the right pulmonary artery. Three loops of 4 to 5 beats were recorded serially with simultaneous electrocardiographic signal recordings and blood pressure measurements with an automatic sphygmomanometer (Welch Allyn, Inc., Skaneateles Falls, NY). The acquired images were obtained in the context of the noninvasive clinical research project cited above, which obtained approval from our institutional ethics board.

#### 2.2.2. Motion Estimation

The motion estimation method that was used to compute mechanical parameters from the AA is similar to the one described elsewhere for B-mode data [14]. However, since the proximity of the AA with the heart and the lungs induces very complex artifactual movements of this artery, a previous rigid registration procedure was then implemented prior to tissue motion estimation.

### 2.3. Statistical Analyses

“SigmaStat” (Systat Software Inc., ver. 3.11.0, 2004) was used for statistical analyses. Data on the effect of aging on the carotid stiffness in rat models of hypertension (HT) were computed with One-Way Analysis of Variance (ANOVA) Kruskal-Wallis' and Dunnett's tests to identify the turning point where the increase in stiffness mainly takes place. Otherwise, One-Way ANOVA Tukey test was used as well to investigate physiological parameters in rat models of HT as to compare post-KD sequelae at the AA level.

## 3. Results

### 3.1. ImBioMark Implementation in Rat Models' Carotid

#### 3.1.1. Stiffness Quantification

**Figure 1:**
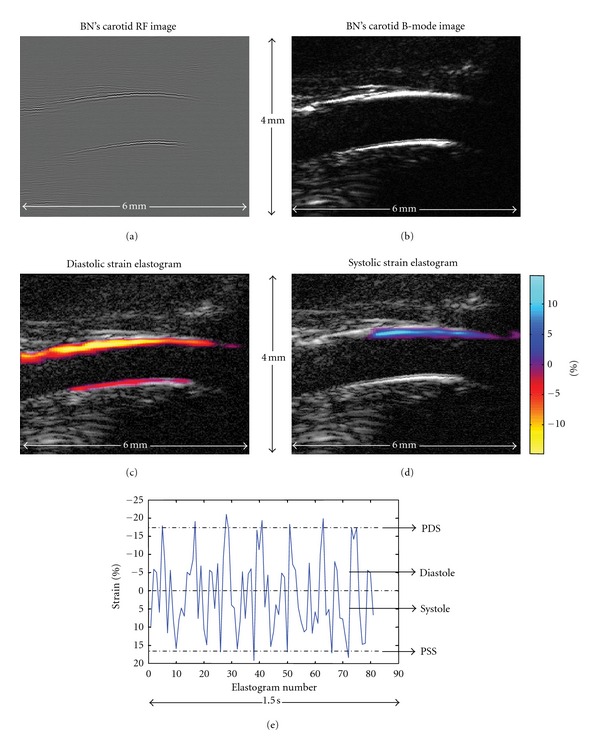
(a) BN's carotid RF image, (b) BN's carotid B-mode image, (c) diastolic strain elastogram that superimposes the B-mode image, (d) systolic strain elastogram that superimposes the B-mode image, (e) MAS curve showing the PDS and PSS parameters that were used as relative measures of stiffness.

Since a high frequency ultrasound transducer (40 MHz) was used, the carotid far wall is most of the times a lot less identifiable than the near one, that is because of signal attenuation with depth. Owing to that, the strain elastograms were analyzed with respect to the near wall.  Figure 1(a) displays a typical RF image of BN's carotid, whereas, for illustrative purposes, Figure 1(b) presents the equivalent B-mode image. Figures 1(c) and 1(d), respectively, exhibit typical systolic and diastolic axial strain distribution images (Δ_*yy*_), also said elastograms, which superimpose the B-mode. In this display, the elastograms were segmented manually and postprocessed with a 5 × 5-pixel median filter. Color bars give strain in percentages. In this configuration, blue is associated with positive strain values, while yellow is indicative of negative values. Systolic axial strain elastograms are characterized by positive strain values, indicating vessel wall compression. Inversely, diastolic strain elastograms are characterized by negative values within the wall, indicating dilation.

As previously described [14], mean axial strain (MAS) was calculated for a 5 × 9-pixel (axial lateral) region-of-interest (ROI) within the near wall of each axial strain elastogram to provide the MAS curve. Such a MAS curve is plotted in Figure 1(e) over 7 consecutive CC. As can be observed in Figure 1(e), peak systolic strain (PSS) and peak diastolic strain (PDS) values are very stable and reproducible. We then propose PSS, which averages PSS values over 3 CC, and PDS, which averages PDS values over 3 CC, as stiffness parameters.

#### 3.1.2. Stiffness as a Function of Aging

One-way ANOVA indicated no significant statistical difference between left and right carotids' strain values, with Δ_*yy*_ = 6.52 ± 3.51% and Δ_*yy*_ = 6.52 ± 3.76%, respectively (*P* = 0.681). *Pooling* data for sides (left/right), no significant statistical difference was found between PDS and PSS, in absolute values, with 6.08 ± 3.06% and 6.93 ± 4.05% strain values, respectively (*P* = 0.189). The mean strain value that is reported to evaluate BN's carotid stiffness as a function of aging was obtained by *pooling* sides (left and right) and CC phase (systole and diastole); the animal population was then virtually increased by a factor of 4.

**Figure 2:**
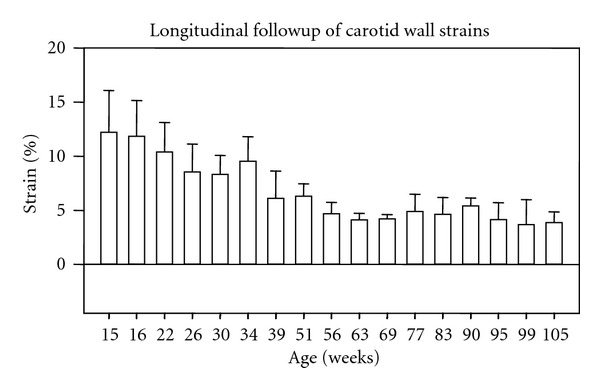
BN's carotid stiffness as a function of aging. Dunnett's test indicated that the increase in stiffness mainly takes place around 40 weeks of age.

Figure 2 plots the interrelationships between BN's carotid stiffness (axial strain) and aging. The strain values stand from 12.24 ± 3.84% at 15 weeks old down to 3.91 ± 0.95% at 105 weeks old. In addition, Dunnett's test indicated that such an increase in the carotid stiffness mainly takes place around 40 weeks of age.

#### 3.1.3. Physiological Parameters

Complementary to ImBioMark, we here report some physiological data. All those measurements, which also took place from animals' 15 up until 105 weeks of age, were recorded along with MicroNIVE data.

**Figure 3:**
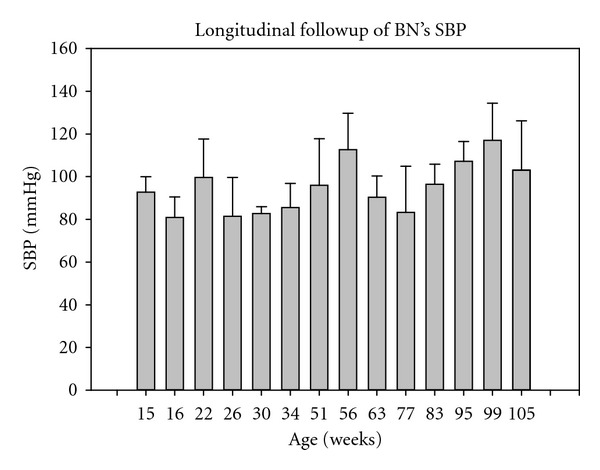
Follow-up of BN's SBP as a function of aging. By removing SBP values at 56 and 99 weeks old, no significant change in SBP was observed, *P* = 0.108.

Figure 3 plots systolic blood pressure (SBP) follow-up as a function of aging. However, SBP monitored at 56 and 99 weeks of age were relatively high with respect to the other measurements. Provided that was basically experimental artefacts, we removed those two experiment SBP values from the statistical analysis. One-way ANOVA then indicated no significant change in SBP along time with an average of 95 ± 18 mmHg, *P* = 0.108. It is to note that BN's SBP usually sets around 120 mmHg. However, because of physiological effects of anaesthesia on the cardiovascular system, such an underestimation was quite expectable.

**Figure 4:**
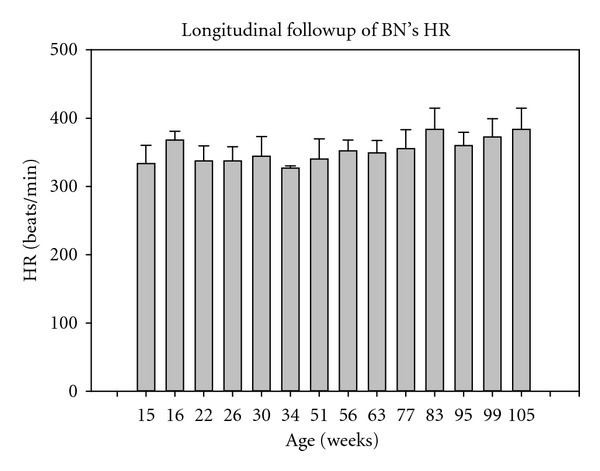
Follow-up of BN's heart rate (HR) as a function of aging. No significant change in HR was observed, *P* = 0.075.

As plotted in Figure 4, we also investigated the follow-up of heart rate (HR) as a function of aging. One-way ANOVA indicated no significant change in HR along time with an average of 351 ± 26 heart-beats/min, *P* = 0.075. Similar to SBP, HR was likely slowed down because of anaesthesia.

**Figure 5:**
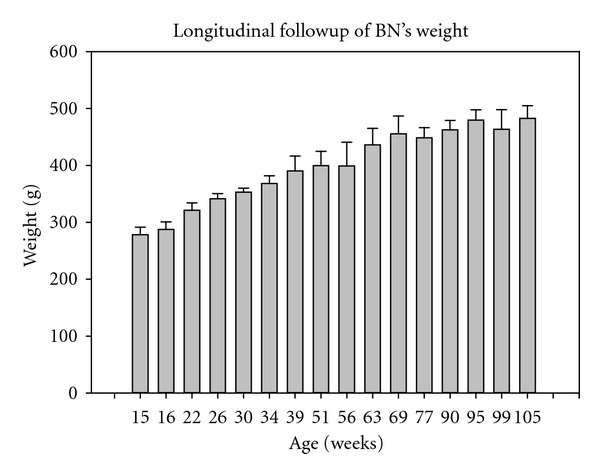
Follow-up of BN's weight as a function of aging. Standing from 278 ± 13 g up to 482 ± 22 g, it was observed to significantly increase with age (*P* < 0.001), but was observed to stabilize from around 60 weeks old (*P* = 0.081).

To complete the physiological part of this longitudinal study, Figure 5 plots BN's weight as a function of aging. As could be expected, the weight significantly increased with age, standing from 278 ± 13 g at 15 weeks old up to 482 ± 22 g at 105 weeks old, *P* < 0.001. Interestingly, the weight was observed to stabilize from around 60 weeks old, *P* = 0.081.

### 3.2. ImBioMark Implementation to Evaluate Post-KD Vasculitis

**Figure 6:**
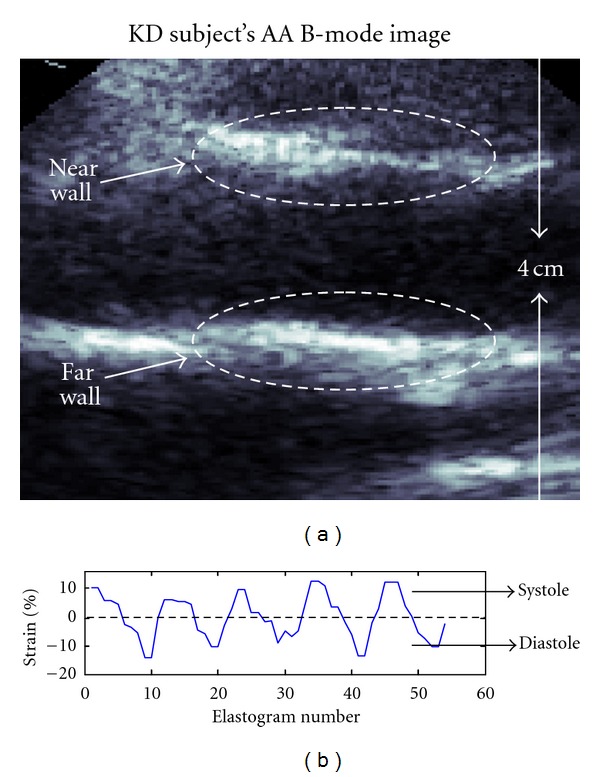
(a) Ascending aorta B-mode image of a KD subject and (b) MAS curve clearly delineating the systolic phase (with positive strain values) and diastolic phase (with negative strain values).

Figure 6(a) displays a typical B-mode image of a KD subject's AA. The artery stiffness was assessed within the near and far walls, respectively, for a 3 × 9-pixel (axial × lateral) ROI. Figure 6(b) plots the MAS curve that was obtained for the far wall of the AA given in Figure 6(a). In this case, we computed mean systolic and mean diastolic strains (MSS and MDS, resp.). Provided there sometimes is an offset between systolic and diastolic strains, a mean strain that is an average of MSS and MDS in absolute values was used to compare KD and KD-free subjects' AA stiffness.

**Figure 7:**
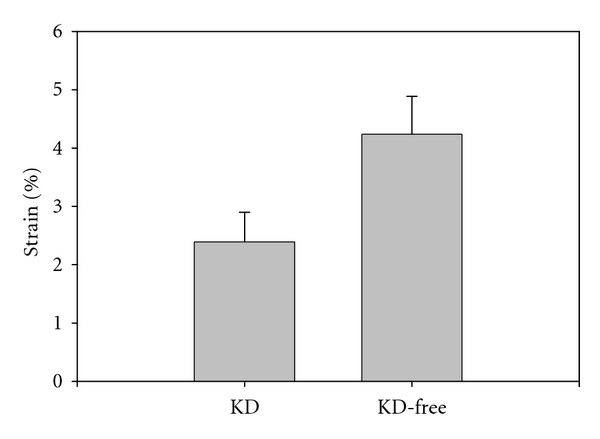
Comparison between KD and KD-free subjects' ascending aorta stiffness. The two groups were found statistically different, *P* < 0.001.

One-way ANOVA indicated no significant statistical difference between near and far walls' strain values, with Δ_*yy*_ = 3.64 ± 0.88% and Δ_*yy*_ = 3.80 ± 1.19%, respectively (*P* = 0.635). The mean strain value that is reported to compare KD and KD-free subjects' AA stiffness was obtained by *pooling* near and far walls. Provided three sets of data were recorded for each subject, we then *virtually* increased our population by a factor of 6.  Figure 7 exhibits a histogram comparing KD and KD-free subjects' AA stiffness. One-way ANOVA indicated that there is a statistical difference between the two groups, with Δ_*yy*_ = 4.24 ± 0.65% (KD-free) and Δ_*yy*_ = 2.39 ± 0.51% (KD), *P* < 0.001.

## 4. Discussion

### 4.1. Hypertension Phenotype with ImBioMark

In a previous paper [14], we have reported data on the effect of salt diet on the carotid artery stiffness of rat models of HT. In the current work, we have investigated the interrelationships between carotid stiffness and aging. We also report some physiological data. We observed that BN's carotid strain decreases with age by a factor close to 3, but with no significant change in SBP.

Provided that the *in vivo* strain measurements were not influenced by physiological parameters (blood pressures and heart rate) and that strain is inversely proportional to stiffness, these results confirm that the carotid artery becomes stiffer with aging. Very interestingly, it is also suggested that this increase in stiffness may take place around a nominal age, that is, 40 weeks old for BN.

The main innovative relevance of MicroNIVE stems from the fact that it would allow *in vivo *investigation of HT, noninvasively and throughout an animal's lifetime. Indeed, despite impressive progress in engineering animal models, such longitudinal studies were not possible before MicroNIVE. To study the pathophysiology of HT, groups of animals are usually sacrificed for tissue or molecular analysis, which eliminates the possibility of understanding regulation *in vivo *in real time as a function of aging, and it also requires a large number of animals.

### 4.2. ImBioMark to Evaluate Post-KD Vasculitis

In this paper, we also report preliminary data on the feasibility and potential of ImBioMark to characterize post-KD vasculitis in humans at the pediatric age. The ascending aorta (AA) was investigated. Whereas the study was limited to 2 KD and to 5 KD-free subjects, data were recorded three times for each subject and were, respectively, analyzed for near, far walls and for systolic, diastolic phases of the cardiac cycle (CC) as to *virtually* increase the data set. In this context, Figure 6 dictates that ImBioMark is reproducible along several consecutive CC. The reproducibility between near and far wall measurements was also statistically validated, *P* = 0.635. In summary, ImBioMark clearly allowed dissociating AA stiffness of KD subjects (Δ_*yy*_ = 2.39 ± 0.51%) versus KD-free (Δ_*yy*_ = 4.24 ± 0.65%), *P* < 0.001.

## 5. Conclusion

In this paper, an imaging-based biomarker (ImBioMark) approach was introduced. The first application reported here addressed hypertension phenotype in rat models. Data reported in a previous study indicated that, in a mean term perspective, high-salt diet induces high blood pressure without remodelling of the carotid artery wall. In the current investigation, we observed that the carotid stiffness increases with aging, but without significant change in blood pressure. These results suggest that an increase in artery stiffness does not necessarily induce high blood pressure or hypertension. However, in return, high blood pressure may, in a long-term perspective, induce arterial wall fatigue thus leading to its remodelling.

Although BN rats are normotensive, we are currently investigating interrelationships between the carotid stiffness and aging in hypertensive rats, namely, salt hypertensive and recombinant inbred models. Because ImBioMark is noninvasive and allows *in vivo* follow-up all along complete animal's lifetime, it seems logical to promote this tool for future investigations in the pathophysiology of hypertension.

In addition, we also reported preliminary data on the potential of ImBioMark to evaluate post-KD vasculitis in humans at the pediatric age. In the light of the very promising results, we plan to further investigate prospectively newly diagnosed KD patients in an observational prospective study. The physiology of the ascending aorta and the related stiffness may lead to new risk stratification of the disease. Furthermore, with the potential of ImBioMark to fully evaluate such a remodelling in the peripheral arteries, we also intend to extend this investigation to larger KD populations studying post-KD vasculitis at the carotid artery level.
